# In situ decoration of Au NPs over polydopamine encapsulated GO/Fe_3_O_4_ nanoparticles as a recyclable nanocatalyst for the reduction of nitroarenes

**DOI:** 10.1038/s41598-021-90514-x

**Published:** 2021-06-11

**Authors:** Saba Hemmati, Majid M. Heravi, Bikash Karmakar, Hojat Veisi

**Affiliations:** 1 Department of Chemistry, Payame Noor University, Tehran, Iran; 2 Department of Chemistry, School of Science, Alzahra University, PO Box 1993891176, Vanak, Tehran, Iran; 3 Department of Chemistry, Gobardanga Hindu College, Gobardanga, India

**Keywords:** Chemistry, Nanoscience and technology

## Abstract

A new and efficient catalyst has been designed and prepared via in situ immobilization of Au NPs fabricated polydopamine (PDA)-shelled Fe_3_O_4_ nanoparticle anchored over graphene oxide (GO) (GO/Fe_3_O_4_@PDA/Au). This novel, architecturally interesting magnetic nanocomposite was fully characterized using different analytical techniques such as Field Emission Scanning Electron Microscopy, Energy Dispersive X-ray Spectroscopy, elemental mapping, Transmission Electron Microscopy, Fourier Transformed Infrared Spectroscopy, X-ray Diffraction and Inductively Coupled Plasma-Atomic Electron Spectroscopy. Catalytic activity of this material was successfully explored in the reduction of nitroarenes to their corresponding substituted anilines, using NaBH_4_ as reducing agent at ambient conditions. The most significant merits for this protocol were smooth and clean catalysis at room temperature with excellent productivity, sustainable conditions, ease of separation of catalyst from the reaction mixture by using a magnetic bar and most importantly reusability of the catalyst at least 8 times without any pre-activation, minimum loss of activity and considerable leaching.

## Introduction

Recently, utilization of engineered and appropriately designed heterogeneous catalysts has attracted much attention and stirred up the interest in synthetic organic chemists. Heterogeneous catalysts have showed superiority over their homogeneous counterparts in terms of their ease of separation from the reaction mixture, mildness of reaction conditions, evading tedious work-up procedures and most importantly, the regeneration and reusability of catalysts without any pre-activations. Moreover, instead of successive runs there occurs no appreciable loss in their catalytic activity and considerable leaching.

Synthetic chemistry has accomplished utmost sophistication in terms of easy handling of chemicals, facile and effective purification of products, ease of separation, avoiding decontamination and efficient reusability of catalysts several times^1–6^. While dealing with the catalyzed synthesis of pharmaceutically active compounds, retrieval of the catalyst is important both from economic and hygienic points of view. In some pharmacological processes, the product should be absolutely free from even trace amount of catalyst based on the US or British pharmacopeia assay^7^. Consequently, the magnetic nanoparticles have emerged as an effective way out concerning the facile procedure of separation simply by using an external magnet^8–17^. Moreover, when the magnetic catalysts were isolated in pure form, they could efficiently be reused several times with reproducible results. The nanometric size, higher surface to volume ratio augments their catalytic activity tremendously due to increase in the surface atoms^18–21^. In this regard, use of nano ferrites (Fe_3_O_4_) has opened a new gateway to synthetic organic chemists. It contains large number of hydroxy groups for further organo-functionalizations in order to shape it as a core–shell moiety. These types of encapsulated structure provide additional stability to the nanoparticles by preventing their tendency towards agglomeration, high thermal stability, resistance to oxidation, corrosion and decreased solubility in organic solvents^22–26^.

Different magnetic polymer composites such as Fe_3_O_4_@polyaniline, Fe_3_O_4_@polypyrrole, Fe_3_O_4_@polydoapmine have previously been synthesized in situ and reported. The high density polar core structure was found being responsible for their high affinity towards the noble metals by the virtue of which their catalytic activity is undisputable^27–31^.

In material science graphene is considered as a remarkable member with a single atom distance across and a densely packed two-dimensional honeycomb like matrix^32^. It brings in several unique properties like high thermal and mechanical stability, exceptional electrical conductivity within credibly large surface area and adsorption ability. Consequently, graphene has recently acquired significant attention for being used as support in the preparation of efficient catalysts^33–38^. In its oxidized form, so called graphene oxide (GO), contains large number of diverse oxygenated functional groups like –OH, –COOH, carbonyl and epoxy on its surface which facilitates the immobilization of different organo-funationalities and metal nanoparticles (MNP) towards the modified solid acid nanocomposites^39,40^. The synergistic effects of the MNP and GO sheet adjoin several extraordinary features to these novel architectured materials thus, undoubtedly could be considered as one of the marvelous effective catalysts of future^41^.

Gold catalysis has been an enthralling research field due to its well-known fascinating characteristics. They exhibit excellent photocatalytic activities under both UV and visible lights^42^. A wide variety of Au materials find applications in the degradation of organo-pollutants, biological transmission electron microscopy, colorimetric DNA sensing, biomedical applications and catalysis^43,44^. Their unique catalytic activity which relied on its negative redox potential, found being much smaller than bulk gold^43–51^.

In continuation to our current research on the precise designing and sustainable development of novel heterogeneous nano materials as effective catalysts^15,18,20,21,52–69^, we thought, it is worthwhile to construct a noble metal adorned magnetically isolable high surface area nanocomposite and examine its catalytic activity. We are specifically interested in nanocatalyzed organic transformations being conducted under green conditions in order to keep the environment safe and clean. We usually try to use water as the most abundant and cheap greenest solvent to carry out the reactions at room temperature conditions^10,70–77^. Reduction of nitroarenes to corresponding amines is one of the elementary but very important reaction having outstanding industrial implications. The aromatic amines are relatively safer chemical and have broad range of synthetic and biological applications like photographic development, synthesis of dye intermediates, optical brightening, corrosion inhibition, anticorrosion lubrication and in agrochemicals, in pharmaceuticals for the preparation of analgesic, antipyretic and other drugs^78–80^. In addition, nitrophenols are recognized as significant organopollutant of water and highly toxic for human and marine lives. They cause severe damage of liver, kidney and central nervous system. The reduced product, the aminophenols are non-toxic and have many other applications^81,82^.

**Scheme 1:**
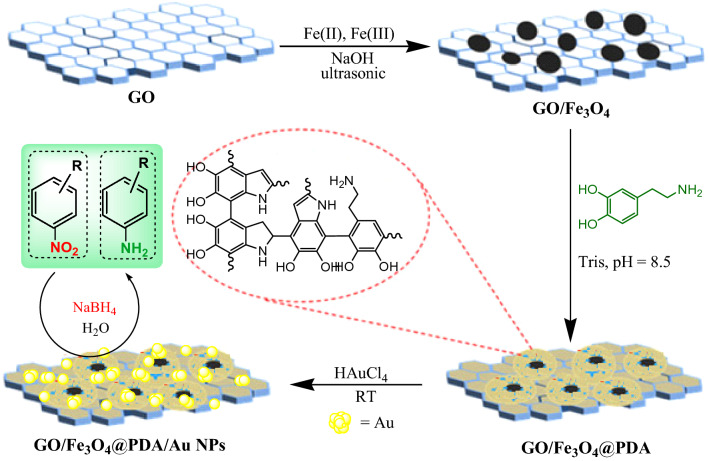
Synthetic scheme of GO/Fe_3_O_4_@PDA/Au nanocatalyst and its application.

Herein, we wish to disclose our experiences in the design and synthesis of a new hybrid nanomaterial, the in situ synthesized Au NP decorated on polydopamine (PDA) functionalized magnetic Fe_3_O_4_ nanoparticles grafted over GO nanocomposite (GO/Fe_3_O_4_@PDA/Au). Its structure was analyzed based on the data obtained using different standard techniques. After unambiguous structural elucidation, we examined its catalytic activity of this novel nanocomposite in the reduction of nitoarenes using conventional reductive agent such as NaBH_4_ under ambient reaction conditions in water (Scheme 1). It is worthy to mention, the reduction of 4-nitrophenol by this catalyst was selected as model reaction and was carefully and prudently monitored using UV–Vis spectroscopy followed by the kinetic study of this reaction.

## Experimental

### Synthesis of GO/Fe_3_O_4_ nanocomposite

GO was prepared following modified Hummer’s method reported elsewhere^83^. A suspension of GO (0.2 g) was ultrasonically treated for 30 min in 100 mL DI H_2_O for exfoliation and then100 mL of 2.5 (M) NaOH solution was added to it under vigorous stirring. In a separate container 0.25 g FeCl_2_·4H_2_O and 0.67 g FeCl_3_·6H_2_O were mixed in 25 mL deoxygenated water with the addition of 0.45 mL conc. HCl. The resulting solution was added dropwise to the alkaline GO suspension and sonicated further. The GO impregnated MNP were isolated from mixture by an external magnet and rinsed with 200 mL DI H_2_O thrice. Finally, it was dried at 40 °C to obtain GO/Fe_3_O_4_ nanocomposite.

### Synthesis of GO/Fe_3_O_4_@PDA/Au nanocomposite

A mixture of 0.5 g GO/Fe_3_O_4_nanocomposite and 0.5 g dopamine was suspended in 500 mL tris buffer (10 mM, pH 8.5) and stirred for 24 h at room temperature in order to polymerize. After completion of reaction, the GO/Fe_3_O_4_@PDA composite was isolated with a magnet and washed with DI H_2_O followed by anhydrous ethanol and dried at 40 °C to afford GO/Fe_3_O_4_@PDA. In the next phase, the GO/Fe_3_O_4_@PDA composite (0.5 g) was ultrasonically dispersed in 100 mL distilled water for 20 min. A solution of 0.04 g HAuCl_4_ in 20 mL water was then added it and refluxed for 12 h. Finally, the GO/Fe_3_O_4_@PDA/Au nanocomposite was magnetically separated and sequentially washed with DI water, ethanol and acetone. The Au content on the catalyst was 4.1 wt%, as determined by ICP-OES.

### Catalytic reduction of nitrobenzene

In the typical synthesis, an emulsion of nitrobenzene (0.003 M) in H_2_O (0.03 mL) was stirred in presence of water suspended GO/Fe_3_O_4_@PDA/Au nanocomposite (0.5 mL, 0.001 g/mL) for 5 min. Then, 0.05 mL aqueous solution of NaBH_4_ (0.001 g/mL) was added to it and stirring was continued. As the reaction progressed, the yellow color of the solution gradually faded out. The entire course of reaction was monitored over UV–Vis spectroscopy. After completion, the catalyst was isolated using a magnet, regenerated and reused in further cycles.

## Results and discussion

### Study of catalyst characterizations

The GO/Fe_3_O_4_@PDA/Au nanocomposite was synthesized following a stepwise post-functionalization approach. At the outset, magnetic graphene oxide (GO/Fe_3_O_4_) was synthesized according the experimental. The NPs were then covered using PDA, being synthesized by in situ polymerization. PDA organizes a suitable polar environment to anchor Au(III) ions over them and reduces to metallic Au NPs promoted by its active catechol and amine functionalities. Scheme 1 depicts the graphic preparative scheme. The as designed nanocomposite was characterized using FT-IR, FESEM, EDX, elemental mapping, TEM, XRD and ICP-OES techniques.

**Figure 1:**
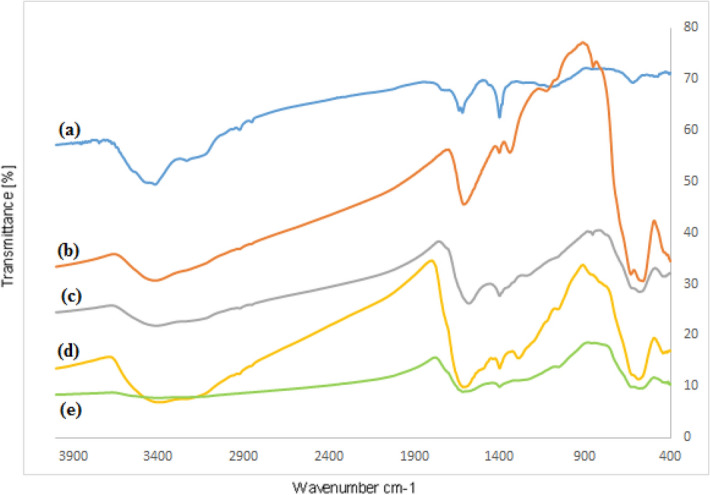
FT-IR spectra of (**a**) GO, (**b**) Fe_3_O_4_, (**c**) GO/Fe_3_O_4_, (**d**) GO/Fe_3_O_4_@PDA and (**e**) GO/Fe_3_O_4_@PDA/Au.

In order to justify the sequential synthesis of GO/Fe_3_O_4_@PDA/Au nanocomposite, FT-IR spectra of all the corresponding intermediates have been presented in Fig. 1. In Fig. 1a the strong broad band observed in the region ∼3100 to 3450 cm^−1^ were attributed to combined C–OH stretching, O–H coupled and the water intercalated stretching vibrations. The C=O stretching vibrations for carbonyl functions and carboxylic acids appeared at 1741 cm^−1^. The absorptions at 1378 cm^−1^ and 1060 cm^−1^ were due to carboxyl O–H and epoxy C–O groups respectively. In Fig. 1b the characteristic absorption peaks at 568 cm^−1^ and 635 cm^−1^ were corresponded to the Fe–O stretching vibrations from Fe_3_O_4_. The overlapping bands from Fig. 1a,b in Fig. 1c clearly indicates the successful incorporation of Fe_3_O_4_ onto GO surface. In the consequent spectrums of GO/Fe_3_O_4_@PDA (1d) and GO/Fe_3_O_4_@PDA/Au (1e), the characteristic band appeared around 2900–2950 cm^−1^was due to C–H stretching and those observed at 3373 and 1586 cm^−1^ were due to N–H stretching. A shifting of N–H band to lower wavenumber region was observed from Fig. 1d–e, probably because of the strong interaction between the N and O groups of dopamine with Au NPs. A further decrease in Fe–O absorption was inferred due to gold attachment (Fig. 1e).

**Figure 2:**
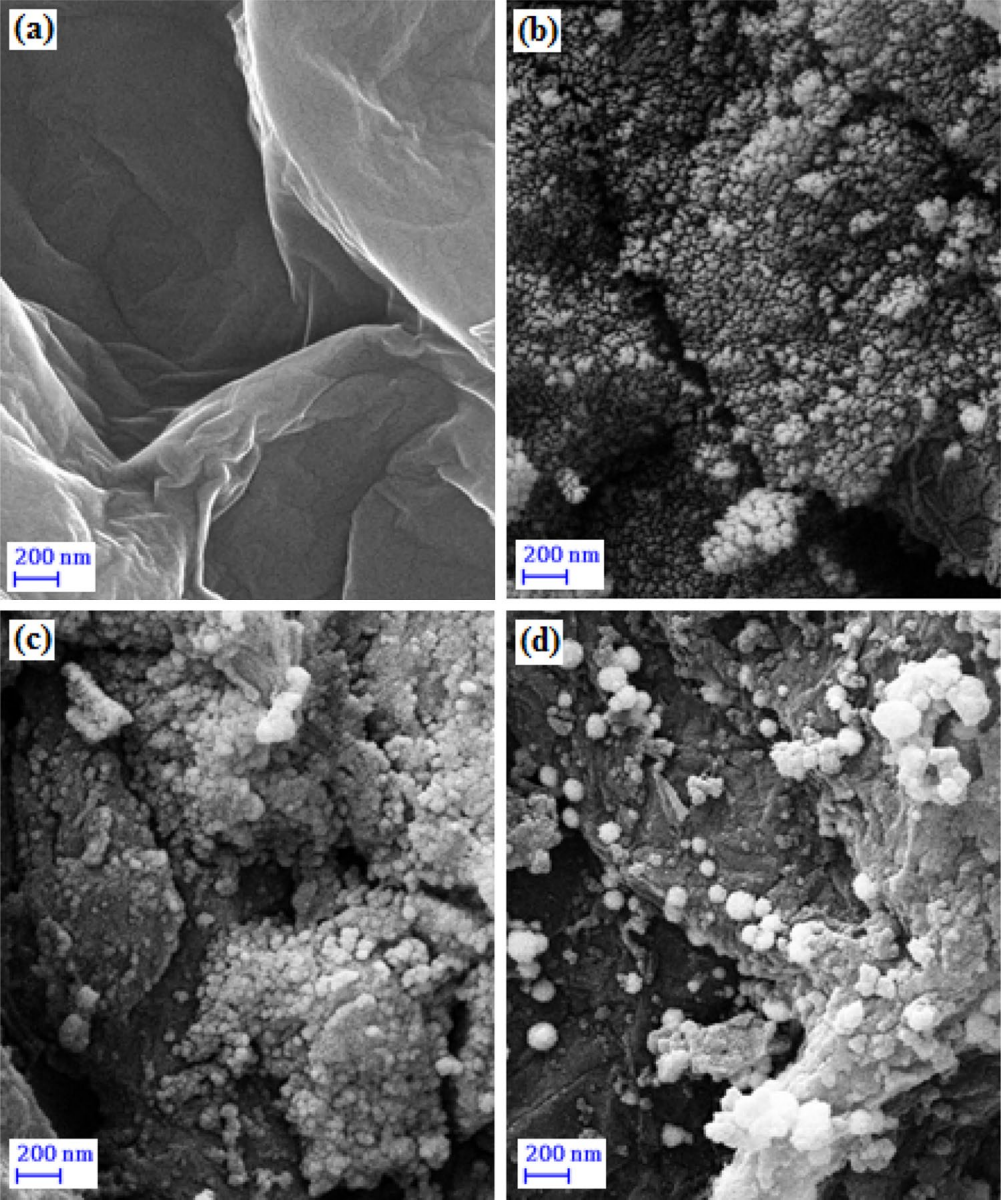
SEM images of (**a**) GO, (**b**) GO/Fe_3_O_4_, (**c**) GO/Fe_3_O_4_@PDA,and (**d**) GO/Fe_3_O_4_@PDA/Au NPs.

The structural morphologies of GO, GO/Fe_3_O_4_, GO/Fe_3_O_4_@PDA and GO/Fe_3_O_4_@PDA/Au samples were determined by FESEM analysis (Fig. 2). GO exhibited a typical folded and wrinkled thin sheet-like appearance (2a). The incorporation of Fe_3_O_4_ NPs into GO surface results an increase in the wrinkles over the surface. It also restrains the stacking of GO planes towards polymeric strictures. The globular magnetite NPs are clearly visible over the GO sheet in Fig. 2b. Due to higher concentration during sampling, it seems somewhat agglomerated. In Fig. 2c the polymeric DA is found to immobilize homogeneously over the GO/Fe_3_O_4_ surface. Au NPs were generated in situ by reduction and capped over PDA and decorated on the GO/Fe_3_O_4_@PDA composite (Fig. 2d).

**Figure 3:**
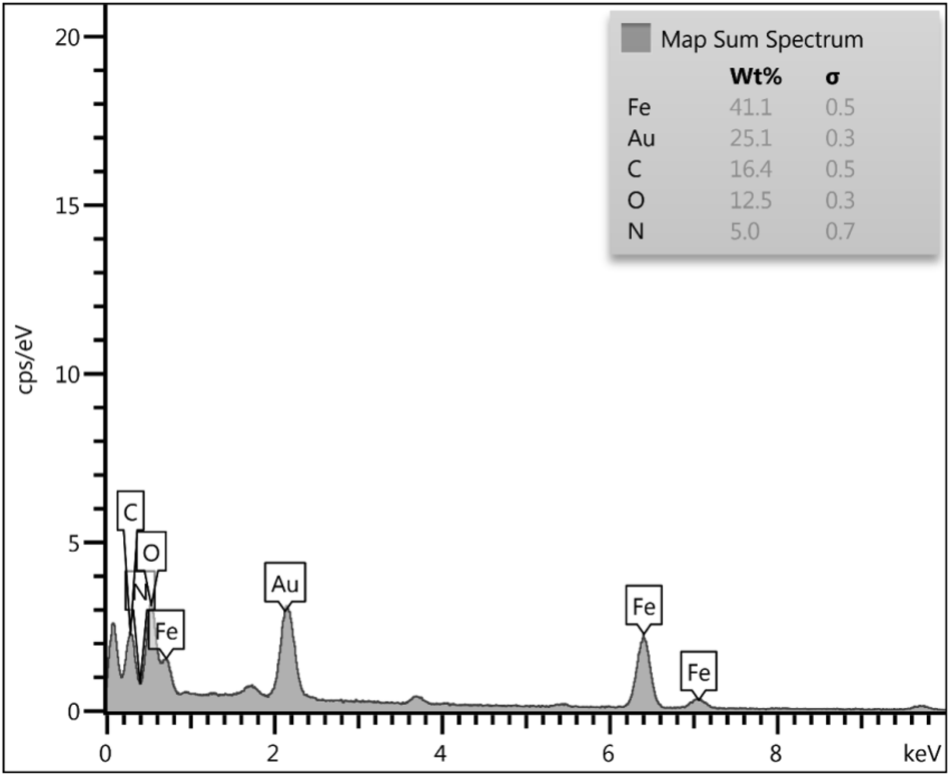
EDX pattern of GO/Fe_3_O_4_@PDA/Au nanocomposite.

The elemental constitution of GO/Fe_3_O_4_@PDA/Au was further confirmed by EDX analysis. Figure 3 displays the EDX profile where Fe and Au are present as metallic component. The C, N and O element validates the PDA and GO attachment in the nanocomposite.

**Figure 4:**
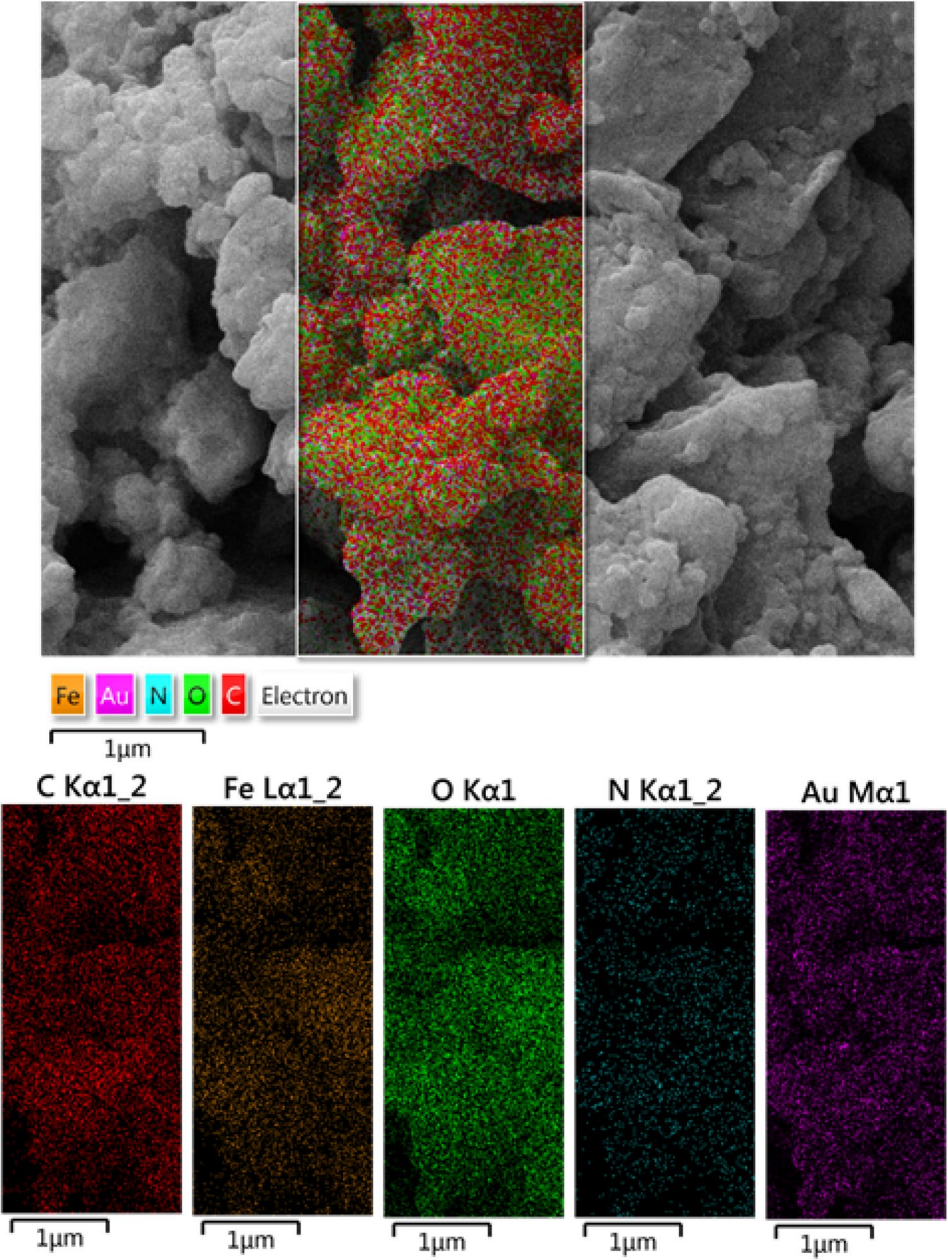
Elemental mapping of GO/Fe_3_O_4_@PDA/Au nanocomposite.

In addition to EDX, elemental mapping of GO/Fe_3_O_4_@PDA/Au nanocomposite was further carried out to study the atomic composition and their distribution over the whole surface. From the SEM image a small surface section is chosen and is analyzed via X-ray dispersion. The results are presented in Fig. 4. The mapping displayed a homogeneous dispersion of C, N, O, Fe and Au atoms in the composite. The occurrence of C, N and O also justifies the organic functionalization over GO.

**Figure 5:**
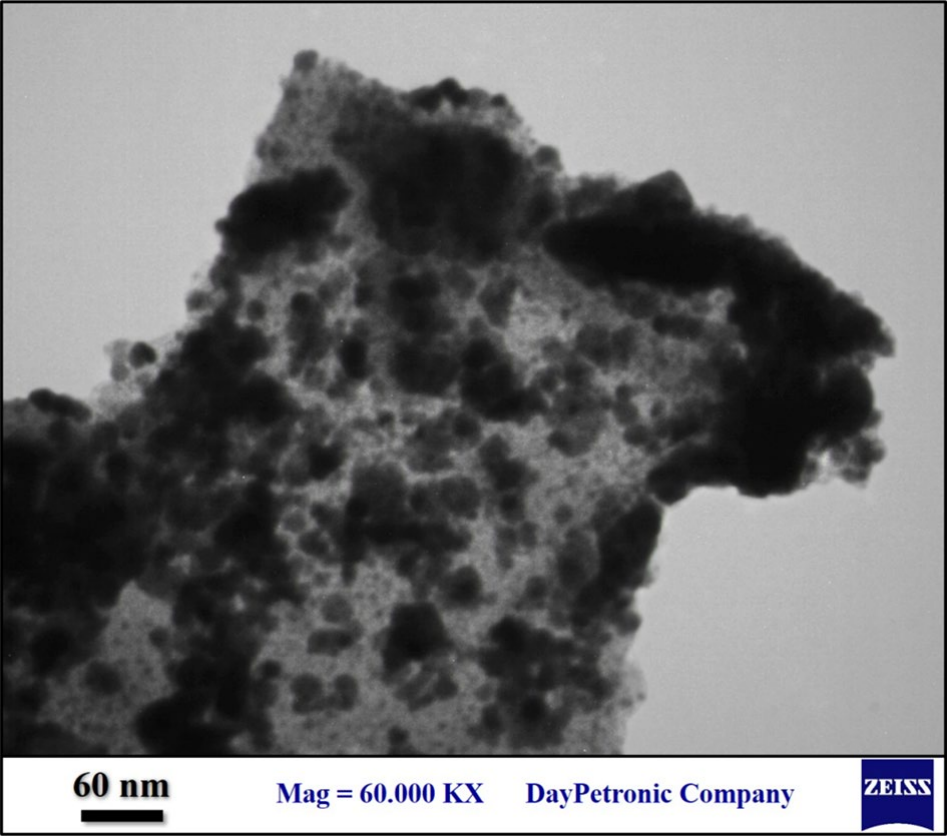
TEM representation of GO/Fe_3_O_4_@PDA/Au nanocatalyst.

More detail of the structural framework was ascertained by TEM analysis of the GO/Fe_3_O_4_@PDA/Au nanocomposite (Fig. 5). It demonstrates the Fe_3_O_4_@PDA/Au conjugates, being seen as dark spots, are uniformly embedded over the transparent GO sheet. However, in some regions it shows the sign of agglomeration. Due to large surface area of GO, individually Au NPs are not visible in the image. Nevertheless, the high dispersion of the active sites led to higher catalytic performance.

**Figure 6:**
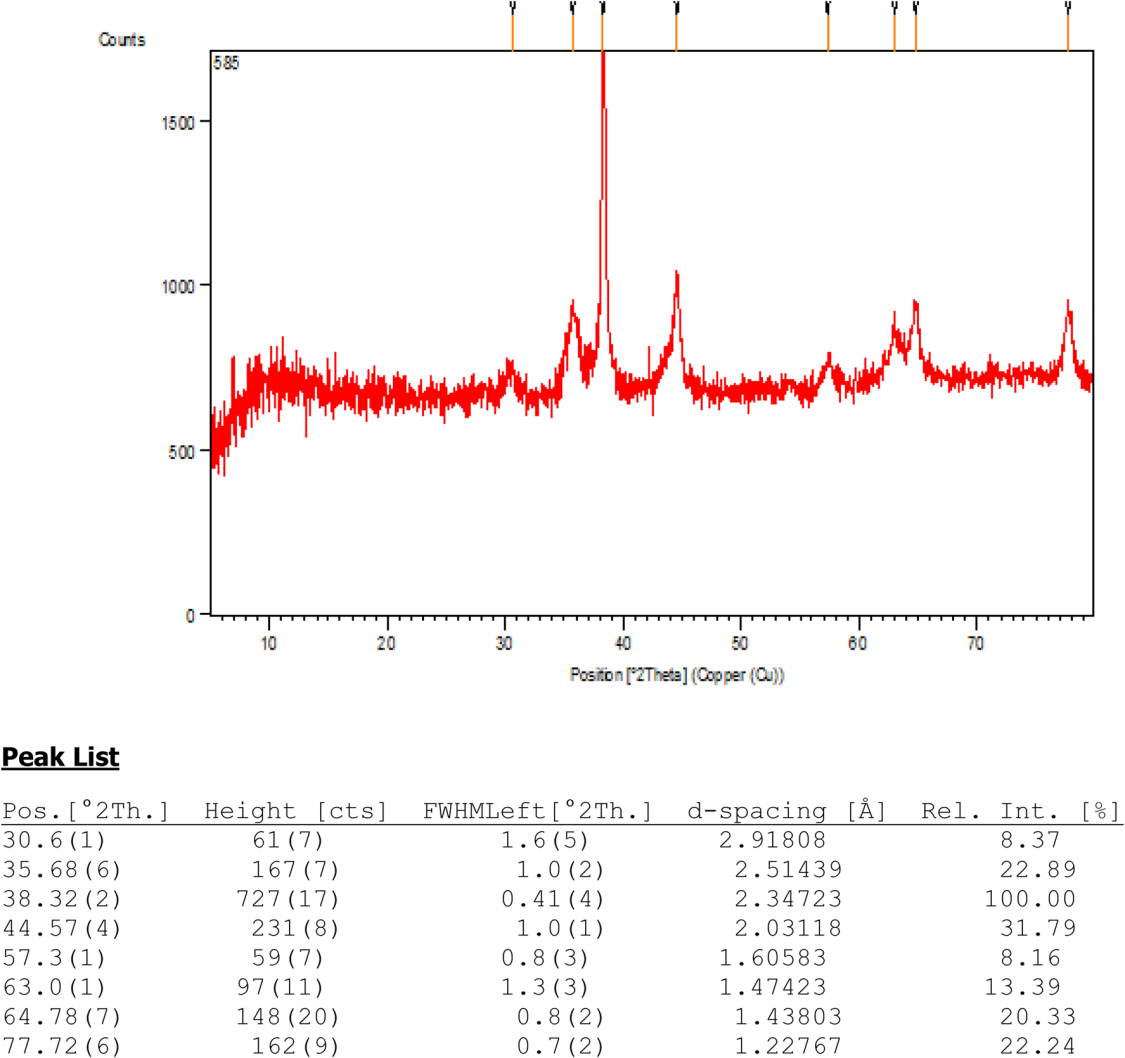
XRD pattern and the peak lists of GO/Fe_3_O_4_@PDA/Au nanocomposite.

To determine the crystallinity of the GO/Fe_3_O_4_@PDA/Au phases, it was analyzed by XRD. Figure 6 displays the single phase XRD profile of the material which indicates that the nanomaterial is a united entity. The corresponding FWHM and d-spacing are also shown there. The Bragg's peaks observed at 30.22°, 35.65°, 43.29°, 53.54°, 57.33° and 62.90°(2θ) are attributed to the reflections on (220), (311), (400), (422), (511), and (440) planes respectively of crystalline Fe_3_O_4_ (JCPDS standard 19-0629 of Fe_3_O_4_)^84^. The pattern indicates that phase morphology of crystalline cubic spinel Fe_3_O_4_ remains intact even after post-grafting. Again, the diffraction peaks at 38.26°, 44.43°, 64.66° and77.61° (2θ) are related to the (110), (200), (220), and (311) planes of crystalline Au phases (JCPDS card no. 04-784)^75^. The decrease in peak intensity from the standard can bepredicted based on the grafting of Au/PDA complex of Fe_3_O_4_over GO.

### Study of catalytic application

**Figure 7:**
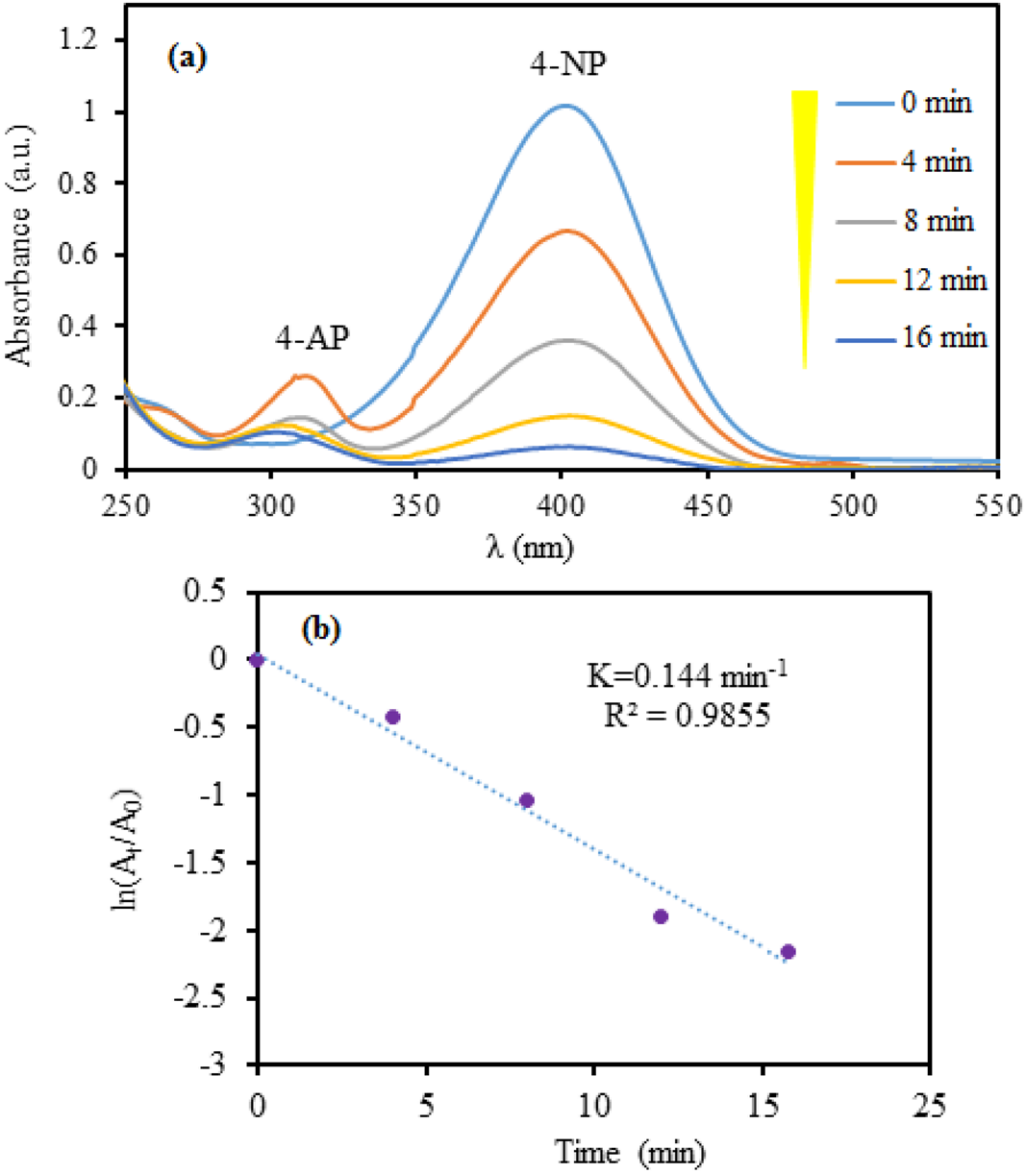
The UV–Vis spectroscopic study in the reduction of 4-NP to 4-AP over GO/Fe_3_O_4_@PDA/Au nanocatalyst.

The catalytic exploration of GO/Fe_3_O_4_@PDA/Au nanocomposite was started in the reduction of 4-nitrophenol as model reaction at room temperature using NaBH_4_as reducing agent. The entire process was quantitatively monitored over a UV–Vis spectrophotometer. Initially, in the absence of catalyst, when NaBH_4_ was added, the pale yellow color was intensified due to the formation of 4-nitrophenate ion and a red shift was identified. The characteristic absorption maxima of 317 nm were shifted to 400 nm. Just after the addition of catalyst, the color as well as peak intensity started diminishing, which indicated the initiation of reduction of 4-NP. Without the addition of catalyst, reaction did not proceed at all. As the reaction progressed, the bell shaped curve, corresponding to λ_max_ 400 nm, gradually flattened and concurrently a new peak was generated at 295 nm due to the formation of 4-AP (Fig. 7a). The reduction was completed in 16 min as visually indicated by the decoloration of solution^85^. A kinetic study for the reaction was also carried out using the spectroscopic data. It represented a linear relationship when − ln (A_t_/A_0_) was plotted against reaction time (t) for the process (Fig. 7b) where A_t_ and A_0_ being the absorbance of 4-NP at time t and 0, respectively. The curve fitted absolutely with pseudo first-order reaction kinetics^86^. The rate constants (k) was obtained from the slope as being 0.15 min^−1^ (R^2^ = 0.985).

The control experiments for the reduction of 4-NP to 4-AP were tested. With the purpose of having optimized catalytic conditions, the experiment was also investigated with bare Fe_3_O_4_ NPs, GO/Fe_3_O_4_ and GO/Fe_3_O_4_@PDA nanocomposites as catalyst keeping other conditions intact. Interestingly, no noticeable transformation was detected even after 2 h, which evidently demonstrates the role of Au NPs being stabilized over GO/Fe_3_O_4_@PDA. The results showed that in both conditions without NaBH_4_ and in the presence of the GO/Fe_3_O_4_@PDA/Au there is no progress in the reduction reaction after 5 h. Also, the reduction reaction was done in the presence of Fe_3_O_4_ NPs, GO/Fe_3_O_4_ and GO/Fe_3_O_4_@PDA nanocomposites, which showed the yield was low and end time of the reduction reaction was longer than GO/Fe_3_O_4_@PDA/Au nanocomposite (Table 1).

**Table 1:** The control experiments for the reduction of 4-NP to 4-AP.

Entry	Catalyst	Time (min)	Conversion (%)
1	Fe_3_O_4_-NaBH_4_	120	55
2	Go/Fe_3_O_4_-NaBH_4_	120	60
3	GO/Fe_3_O_4_@PDA-NaBH_4_	120	50
4	GO/Fe_3_O_4_@PDA/Au-NaBH_4_	16	98
5	GO/Fe_3_O_4_@PDA/Au	3000	N.R

Reaction conditions: 0.3 mL of 0.003 M 4-NP, 0.05 mL of 1 mg mL^−1^catalyst, and 0.3 mL of 1.2 (M) NaBH_4_ solution in water.

Now, in order to generalize, we further extended our catalytic explorations with our developed catalyst in the reduction of several other nitroarenes, being monitored over UV–Vis spectrometry and the results have been documented in Table 2 Notwithstanding the type and location of substituent (1-Cl, 2/3/4-CH_3_, 2/4-OH, 2/3/4-NH_2_, 2-OCH_3_) in the aryl ring, all the substrates were highly compatible in the reaction conditions and afforded excellent conversions. The productivity was in the range of 90–98%. Notably, the electron rich nitroanilines (Table 2, entries 11–13) were found to undergo reduction at a relatively faster rate (10–15 min) as compared to electron deficient chloro (Table 2, entry 2) or dinitro substrates (Table 2, entry 6) (90 min). Among the nitrophenols, the reduction of 2-susbtituted nitroarene was sluggish, might be due to field effect or spatial electron inhibition. A wide number of nitroarenes have been found to be compatible at the developed conditions resulting outstanding yields in very short reaction times. Due to the innate ferromagnetism, the catalyst was easily isolated using a magnetic stick and reused for several times (Table 2, entry 8).

**Table 2:** Reduction of various nitrobenzenes using GO/Fe_3_O_4_@PDA/Au catalyst.

Entry	Nitroarene	Time (min)	Conversion (%)
1	Nitrobenzenes	30	98
2	2-Nitrochloro-benzene	90	95
3	4-Nitrotoluene	20	96
4	2-Nitrotoluene	45	95
5	3-Nitrotoluene	60	90
6	2,4-Dinitrotoluene	90	98
7	4-Nitrophenol	16	100
8	2-Nitrophenol	60	95
9	2,4-Dinitrophenol	30	98
10	2-Nitroanisole	15	98
11	4-Nitroaniline	10	98
12	2-Nitroaniline	12	98
13	3-Nitroaniline	15	96

Reaction conditions: 0.3 mL of 0.003 M nitrobenzene, 0.05 mL of 1 mg mL^−1^catalyst, and 0.3 mL of 1.2 (M) NaBH_4_ solution in water.

### Discussion of reaction mechanism

**Scheme 2:**
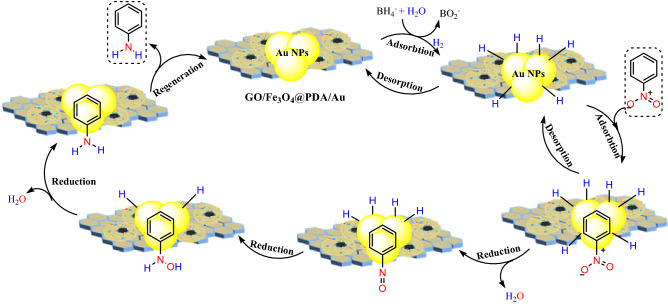
The catalytic mechanism for reduction of nitrobenzene GO/Fe_3_O_4_@PDA/Au catalyst.

For mechanism discussion, more attention should be paid to the catalytic kinetics. The catalytic process is a complex process, which involves the diffusion process, adsorption process, catalyst wetting angle and etc.^87–89^. The plausible catalytic pathway for the reduction of nitrobenzene over GO/Fe_3_O_4_@PDA/Au nanocomposite in presence of NaBH_4_ can be explained based on the Langmuir–Hinshelwood model^90,91^. Initially, the BH_4_^–^ions get adsorbed on the catalyst surface and generate hydride ions (H^–^) in situ towards the formation an Au-hydride complex. Subsequently, the substrate nitrobenzene (NB) also approaches the nano Au surface. The adsorption of both H^−^ and NB occurs reversibly following Langmuir isotherm. Then, interfacial electron transfer occurs from hydrides to NB. The rate of electron transfer is proportional to the conversion rate. The reduction pathway to aniline involves two fast intermediate steps via nitrosobenzene and hydroxylamine^92,93^. A slow hydro-deoxygenation step is followed thereafter in the reduction from hydroxylamine to aniline, being considered as the rate determining step. Finally, desorption of aniline takes place from the catalyst surface to make it free for a new cycle. These whole process of diffusion of reactants, adsorption/desorption equilibria are very facile over the Au catalyst (Scheme 2).

### Reusability and leaching test of GO/Fe_3_O_4_@PDA/Au nanocomposite

**Figure 8:**
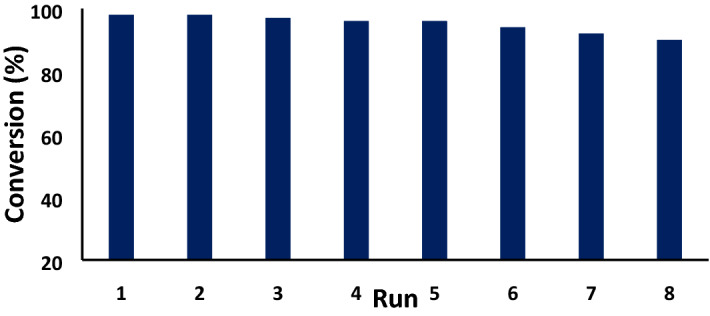
Reusability GO/Fe_3_O_4_@PDA/Au in the reduction of 4-NP.

**Figure 9:**
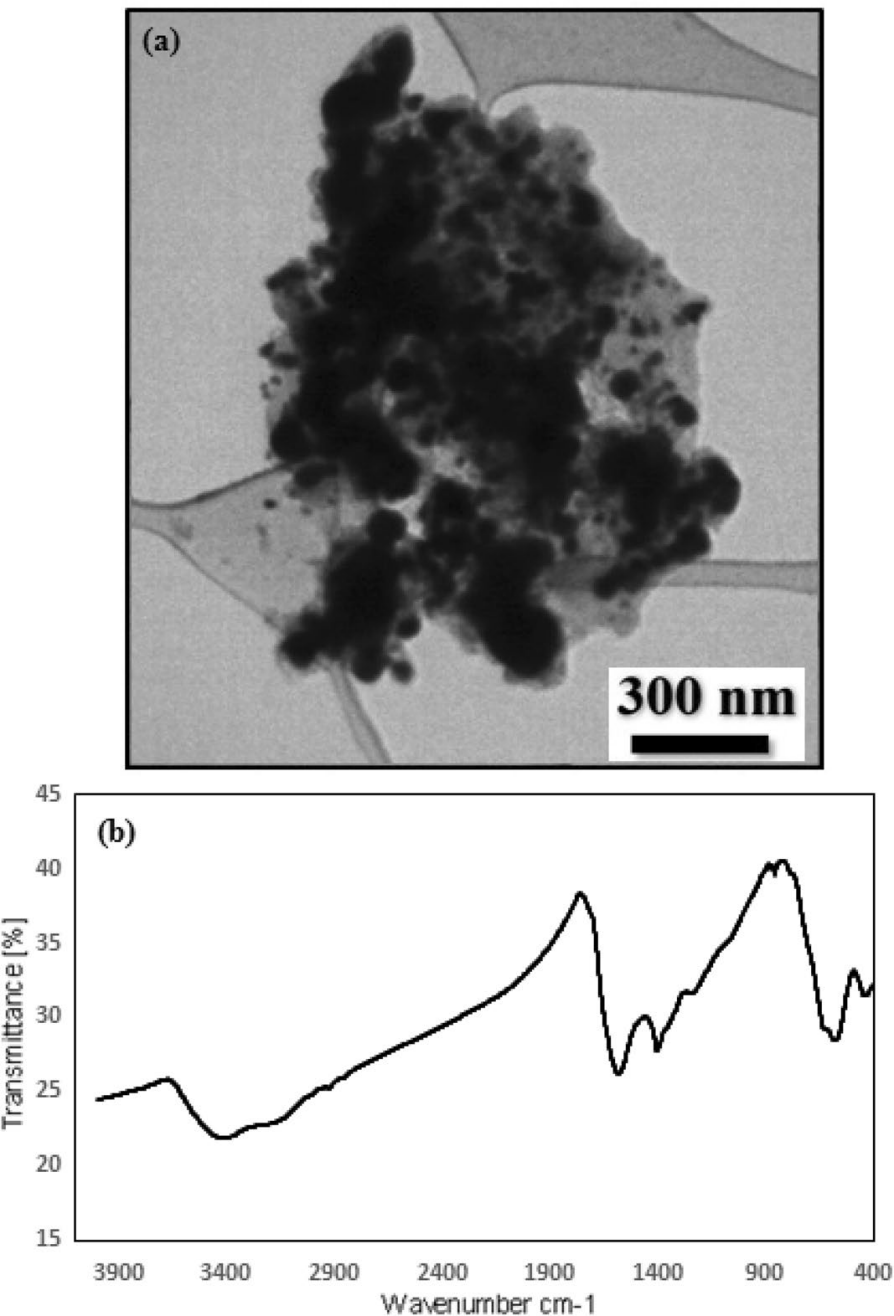
TEM image (**a**) and FT-IR spectrum (**b**) of GO/Fe_3_O_4_@PDA/Au catalyst after 8th run.

In sustainable catalytic methodology, facile isolation, regeneration and reusability of catalyst is an indispensable task. After the successful demonstration of catalytic efficiency of GO/Fe_3_O_4_@PDA/Au nanocomposite in the reduction of nitroarenes, it was the turn to prove those said criteria. Due to the strong inner ferromagnetic core, it was isolated totally with ease from the reaction mixture 4-NP reduction by using an external magnet. The catalyst was then washed with aqueous ethanol, dried and recycled for 8 times with no appreciable loss in activity (Fig. 8). To emphasize the fact, a TEM analysis was conducted with the reused catalyst after 8th run. Amusingly, it retained its structural morphology as initial, which can be seen from Fig. 9a. Also, a FT-IR spectrum for reused catalyst after 8th run (Fig. 9b) shown the same signals without changes in functionality with fresh catalyst. Hence, significant stability of the material validates its outstanding reusability. A leaching test was carried out as well in order to prove the robustness of our catalyst. After the isolation of the catalyst from reaction mixture, an ICP-AES analysis was performed with the reaction filtrate. It was gratifying to ensure that only a marginal amount of Au has been leached out. After the 7th cycle, the Au content in the nanocomposite was greater than 90%. The slight decrease in yield in the 8th cycle is probably due to this loss of Au and the adsorption of product (4-AP) over the catalyst surface^94^.

### Distinctiveness of our result

To prove the uniqueness of our devised protocol, we justified our results in the reduction of 4-NP with some previously reported methods which have been shown in Table 3. As can be seen, the GO/Fe_3_O_4_@PDA/Au catalyst definitely has superiority over others in terms of rate constant.

**Table 3:** Comparison of catalytic results between GO/Fe_3_O_4_@PDA/Au with some reported methods in the reduction of 4-NP.

Entry	Catalyst	T (K)	k (s^−1^) × 10^–3^	References
1	Ag-DENs	298	7.0	^95^
2	AgNPs-rGO	298	0.44	^96^
3	Ni@Au/KCC-1	293	4.3	^97^
4	Ag/C	298	5.4	^98^
5	Ag/Nanosilica	306	1.1	^99^
6	Ni/RGO	298	6.7	^100^
7	Au/graphene	298	3.17	^101^
8	Carbon@Au	298	5.43	^102^
9	Fe_3_O_4_/Ag@NFC	298	3.3	^103^
10	GO/Fe_3_O_4_@PDA/Au	298	14.4	This work

## Conclusions

In summary, we have prepared an Au NP implanted PDA coated magnetic GO nanocomposite (GO/Fe_3_O_4_@PDA/Au). The material was prepared following stepwise post-synthetic approach involving the in situ green reduction of Au (III) ions, without using any harsh conditions. PDA acts as the green reductant as well as the stabilizer of Au NPs. The enormous surface area of GO was exploited for grafting the Au(0)/PDA@Fe_3_O_4_ complex. After full characterization of this novel catalyst using different standard techniques, its catalytic activity was examined towards the reduction of nitroarenes to their corresponding amines. Initially, 4-nitrophenol was selected as a model compound and treated with sodium borohydride in the presence of aforementioned composite at room temperature. The reduction proceeds smoothly leading to the formation of p-hydroxyaniline, a useful starting material for production of acetoaminophen (paracetamols), an over –counter analgesic medication. Then, the as synthesized nanocatalyst was employed in the reduction of a wide range of nitroaromaticssusingNaBH_4_ as the hydrogen source with outstanding conversions and great selectivity. After the completion of the reaction, the catalyst was separated easily, just by using a magnet bar and without any pre-activation were reused for 8successive cycles with almost consistent reactivity. The material was stable enough towards leaching as confirmed by ICP-AES analysis. Its excellent catalytic performance is assumed to be due to synergistic bridged interaction between Au(0),Fe_3_O_4_ and GO sheets, facilitating the faster electron transport to the substrate towards the reduction.
